# Long-term results of type B aortic dissection patients with tumor after endovascular repair or optimal medical therapy: a single—center and retrospective cohort study

**DOI:** 10.1186/s12893-021-01327-8

**Published:** 2021-08-18

**Authors:** Taiping Liang, Hongqiao Zhu, Lei Zhang, Shuangshuang Li, Xiaomin He, Kaiwen Zhao, Zaiping Jing, Jian Zhou

**Affiliations:** Department of Vascular Surgery, Changhai Hospital, Naval Medical University, Shanghai, 200433 China

**Keywords:** Aortic dissection, Thoracic aortic endovascular repair, Optimal medical treatment, Cancer, Outcomes

## Abstract

**Background:**

The effect of thoracic endovascular aortic repair (TEVAR) for acute Type B aortic has been confirmed, However, when patients with malignant disease suffer from acute type B aortic dissection (ATBAD), the effect of TEVAR intervention is still unclear.

**Methods:**

ATBAD patients were identified from electronic medical records between 2009 and 2019. The 5 year overall and aortic-disease free survival rates were analyzed and compared between the two groups.

**Results:**

Of the 40 enrolled patients, 27 (67.5%) received TEVAR and 13 (32.5%) received OMT. The baseline characteristics of the two groups were not significantly different. Kaplan‒Meier survival curve showed that the 5 year overall survival and 5 year aortic-disease free survival of the TEVAR group were better than those of the OMT group. The Cox proportional hazard model with unadjusted risk showed an 83.0% decrease in 5 year overall mortality (HR, 0.17; 95% CI, 0.05–0.56) and a lower aortic-disease related risk (HR, 0.08; 95% CI, 0.02–0.39) in TEVAR group compared to OMT group. After adjusted for age, gender, smoking, drinking and comorbidities (diabetes mellitus, hypertension and coronary artery diseases), the hazard ratio of 5 year overall mortality was 78.0% lower (HR, 0.22; 95% CI, 0.06.0.81) and the risk of aortic-disease related mortality was 93.0% lower (HR, 0.07; 95% CI, 0.01–0.61) in TEVAR group compared to OMT group. In the cohort stratified by age, sex, the risk of the 5 year overall or aortic-disease related mortality in TEVAR group was relatively reduced compared to OMT group.

**Conclusions:**

Compared to OMT, TEVAR improves the 5 year overall and aortic-disease free survival rates in the cohort of ATBAD patients with a single type of malignant tumors.

## Background

The effect of thoracic endovascular aortic repair (TEVAR) for acute Type B aortic has been confirmed, However, when patients with malignant disease suffer from acute type B aortic dissection, the effect of TEVAR intervention is still unclear. So, we conducted this study to evaluate the long-term estimates of 5 year overall and aortic-disease free survival in ATBAD patients with a single tumor type who underwent the thoracic endovascular repair (TEVAR) or optimal medical treatment (OMT).

## Introduction

Acute aortic dissection (AD) is a fatal but relatively rare disease, while Cancer has become the main cause of death in many countries. [1–3] The advances in cancer therapies have led to increasing life expectancy, therefore, the population of AD patients in cancer survivors is increasing. Currently, thoracic endovascular repair (TEVAR) and optimal medical treatment (OMT) are recognized as the conventional treatments for acute type-B aortic dissection (ATBAD). [4, 5] However, when aortic dissection occurs in a patient with a malignant tumor, the management of these two diseases becomes more challenging. Firstly, inflammatory factors of tumor and chemotherapy drugs are likely to promote the development of dissection and increase the risk of rupture. [6, 7] Secondly, patients with malignancy are often older and have more complications, meanwhile, the hypercoagulability state of cancer complicates the endovascular therapy of AD [8]. Therefore, when treating such patients, physicians often face the dilemma of choosing between TEVAR or OMT. There is limited evidence-based guidance in this cohort, which further adds to the clinical dilemma. [9, 10] This study reviewed the hospitalization and follow-up data of ATBAD patients with cancers in our center from February 2009 to February 2019. We sought to summarize this cohort of patients, analyze their characteristics, treatment patterns, clinical outcomes, and find out whether TEVAR treatment has a certain guiding effect on the benefits and treatment of these patients. Here, we report our findings for this special sub-population.

## Methods

### Patient selection

From 2009 to 2019, A total of 3631 AD cases were diagnosed and treated in our hospital. All patients with ATBAD were identified retrospectively based on the screening of the hospital admission notes with the diagnostic code. 3 individuals were excluded as their AD was secondary to traumatic injury. 28 individuals diagnosed with Marfan syndrome were also excluded. Through further screening, the study obtained 40 cases of patients with malignant tumors and ATBAD. The following inclusion criteria were employed: (1) the patient was diagnosed with ATBAD and malignant disease, requiring treatment, (2) ATBAD and malignant disease were diagnosed at the same time, or (3) malignant disease was diagnosed before ATBAD. Informed consent was obtained from all patients, and this project was approved by the ethical committee of changhai hospital.

### Data collection

For all eligible patients, clinical characteristics and computed tomography angiography scans were retrospectively reviewed. The characteristic data included patient sex, age, smoking, past medical history, tumor types, metastasis, dissection anatomical structure, and treatment. The primary outcome was overall survival at 5 years, and the secondary endpoint was aortic-disease related mortality. Aortic-disease related events were defined as aortic rupture, cardiac complications, and/or organ failure caused by dissection. The final survival state was determined based on the review of the medical case records or telephonic follow-up after discharge. According to the different treatment methods received during hospitalization, patients were divided into TEVAR and OMT groups.

### Statistical analysis

The mean (± SD) was used to describe continuous variables; absolute numbers and percentage frequencies were used for categorical factors. For continuous variables, differences between the groups were evaluated using a two-sample *t*-test or the non-parametric Mann‒Whitney *U* test, depending on the distribution of the variables. Categorical variables were compared using the Fisher exact test or *χ*^2^ test. Time-to-event curves were calculated using the Kaplan–Meier method and compared using the log-rank test. All tests were two-tailed, and *P* < 0.05 was considered significant. R software version 3.5.2 was used to perform the analyses.

## Results

### Base characteristics

A total of 40 patients were admitted to the emergency unit with ATBAD and concomitant malignant diseases between January 2009 and January 2019. Of the 40 enrolled ATBAD patients, 27 (67.5%) received TEVAR and 13 (32.5%) received OMT. The mean and median follow-up durations were 2.7 and 1.8 years, respectively. The baseline characteristics of the patients are shown in Table 1, and there were no significant differences (all *P* > 0.05).

**Table 1:** Baseline characteristics of patients treated with BMT and TEVAR

Treatment	Overall	OMT	TEVAR	*P* value
		N = 13	N = 27	
Age	64.56 ± 9.93	66.23 ± 10.50	63.74 ± 9.74	0.44
Male gender	36 (90.00%)	11 (84.62%)	25 (92.59%)	0.43
Smoking	8 (20.00%)	3 (23.08%)	5 (18.52%)	0.74
Drinking	3 (7.50%)	1 (7.69%)	2 (7.41%)	0.97
Comorbidities				
Hypertension	28 (70.00%)	8 (61.54%)	20 (74.07%)	0.47
Diabetes mellitus	5 (12.50%)	2 (15.39%)	3 (11.11%)	0.99
Coronary artery disease	3 (7.50%)	0 (0.00%)	3 (11.11%)	0.54
Stroke	2 (5.00%)	1 (7.69%)	1 (3.70%)	0.99
Malignant diseases				0.53
Prostate cancer	1 (2.50%)	0 (0.00%)	1 (3.70%)	
Thyroid cancer	3 (7.50%)	0 (0.00%)	3 (11.11%)	
Lung cancer	5 (12.50%)	1 (7.69%)	4 (14.82%)	
Pancreatic cancer	3 (7.50%)	1 (7.69%)	2 (7.41%)	
Bladder cancer	2 (5.00%)	0 (0.00%)	2 (7.41%)	
Gastric cancer	5 (12.50%)	1 (7.69%)	4 (14.82%)	
Sarcoma	2 (5.00%)	1 (7.69%)	1 (3.70%)	
Nasopharyngeal carcinoma	2 (5.00%)	2 (15.39%)	0 (0.00%)	
Cholangiocarcinoma	3 (7.50%)	2 (15.39%)	1 (3.70%)	
Rectal cancer	5 (12.50%)	2 (15.39%)	3 (11.11%)	
Renal cancer	5 (12.50%)	2 (15.39%)	3 (11.11%)	
Liver cancer	4 (10.00%)	1 (7.69%)	3 (11.11%)	
Endstage of cancer	4 (10.00%)	2 (15.39%)	2 (7.41%)	0.58

*OMT* optimal medical treatment, *TEVAR* thoracic endovascular repair

### Distribution of malignant diseases and stratified analysis

**Fig. 1:**
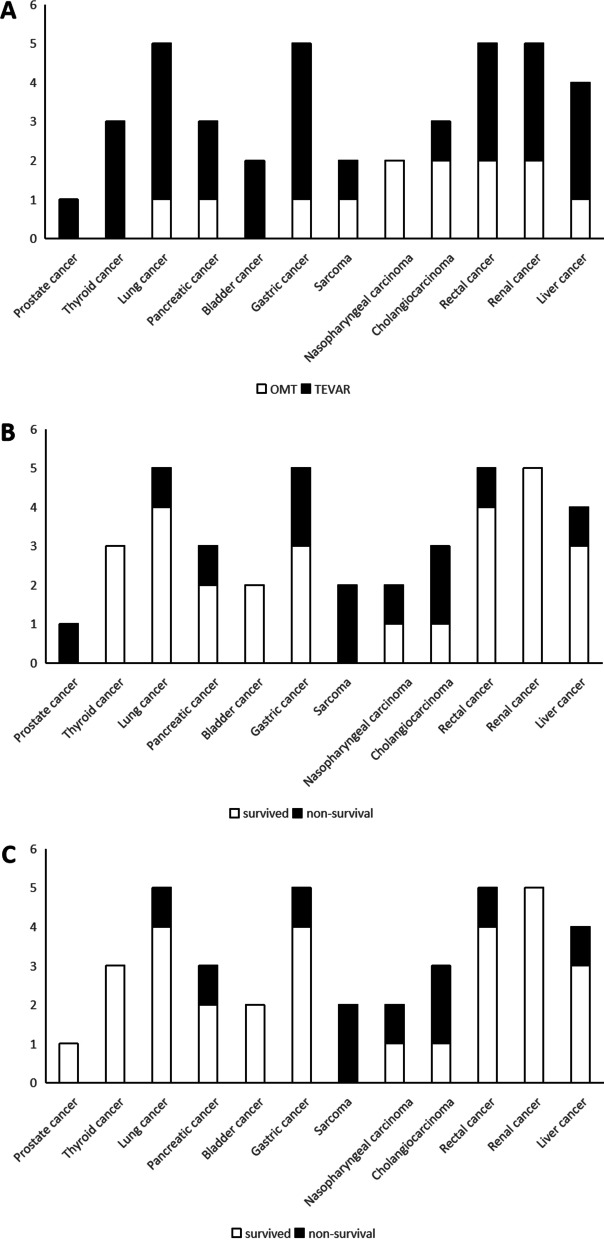
Distribution of malignant diseases in the patients (n = 40), stratified according to **A** choices of treatment, **B** 5 year overall survival, **C** 5 year aorta-related survival

**Fig. 2:**
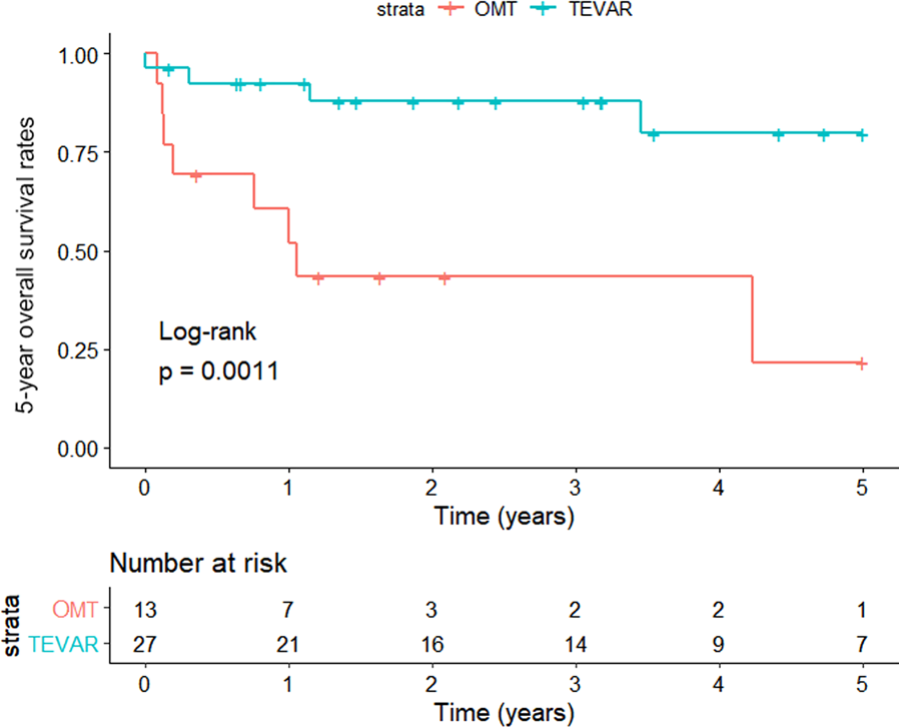
Kaplan–Meier estimates of 5 year overall cumulative survival rate in OMT and TEVAR groups; P = 0.0011 by log-rank test

**Fig. 3:**
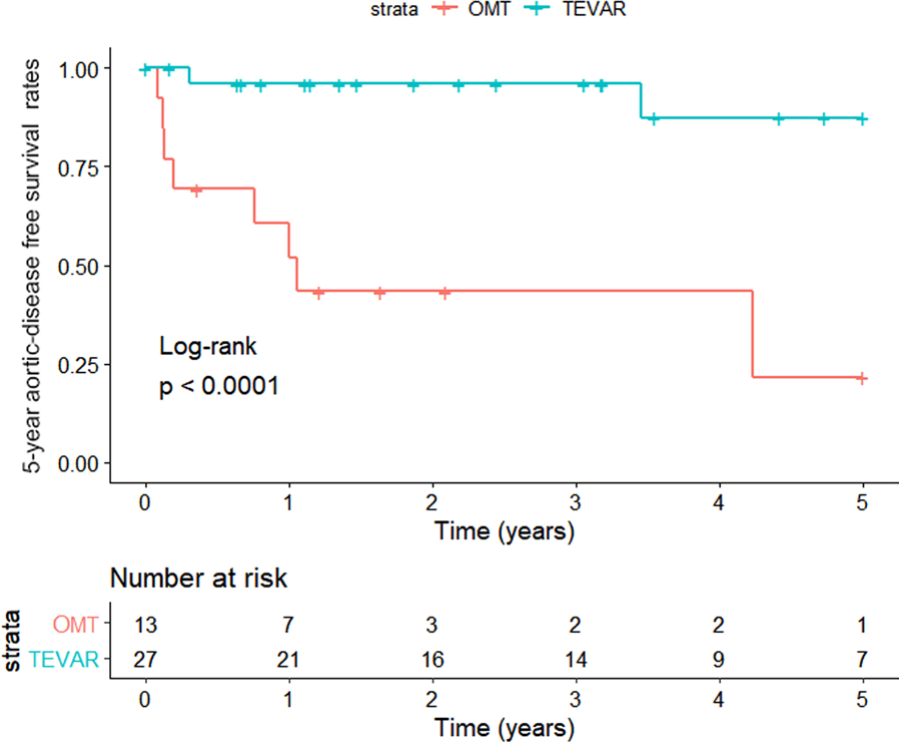
Kaplan–Meier estimates of 5 year aorta-related cumulative survival rate in OMT and TEVAR groups; P < 0.0001 by log-rank test

A total of 40 enrolled ATBAD patients had malignant tumors, and no patient suffered from two or more malignant tumors at the same time (Fig. 1A). The 5 year overall survival status and 5 year aortic-disease related survival status were shown in Fig. 1B and Fig. 1C, respectively. As shown in the Kaplan‒Meier survival curve, the TEVAR group had better 5 year overall survival (Fig. 2) and 5 year aortic-disease free survival (Fig. 3) than the OMT group.

### Association of the treatment with the clinical outcomes

The Cox proportional hazard model with unadjusted risk showed that the TEVAR group exhibited an 83.0% decrease in 5 year overall mortality (HR, 0.17; 95% CI, 0.05–0.56) compared to the OMT group. Similarly, the TEVAR group was associated with a lower aortic-disease related risk than the OMT group (HR, 0.08; 95% CI, 0.02–0.39) (Table 2). Further, in Cox proportional hazard analysis for the adjusted model 1, age and sex were determined to be confounders (Table 2). Results showed that for the TEVAR group, the hazard ratio of 5 year overall mortality was 82.0% lower (HR, 0.18; 95% CI, 0.05.0.61) and the hazard ratio of aortic-disease related mortality was 92.0% lower (HR, 0.18; 95% CI, 0.02.0.41) than that of the OMT group (Table 2). In the adjusted model 2, age, sex, smoking, drinking, and comorbidities (diabetes mellitus, hypertension, and coronary artery diseases) were determined to be confounders (Table 2). Specifically, in model 2, the hazard ratio of 5 year overall mortality was 78.0% lower for the TEVAR group (HR, 0.22; 95% CI, 0.06.0.81) compared to that for the OMT group. For the TEVAR group, the risk of aortic-disease related mortality was 93.0% lower (HR, 0.07; 95% CI, 0.01–0.61) than that for the OMT group (Table 2).

**Table 2:** Unadjusted and adjusted sub-hazard ratio for 5 year mortality according to treatment

Events	Treatment	Unadjusted	Adjusted model 1†	Adjusted model 2‡
		HR (95% CI)	HR (95% CI)	HR (95% CI)
Aorta-related adverse events	OMT	Reference	Reference	Reference
	TEVAR	0.08 (0.02, 0.39)	0.08 (0.02, 0.41)	0.07 (0.01, 0.61)
All cause death	OMT	Reference	Reference	Reference
	TEVAR	0.17 (0.05, 0.56)	0.18 (0.05, 0.61)	0.22 (0.06, 0.81)

^†^Model 1 adjusted for age and gender. ^‡^Model 2 adjusted for age, gender, smoking, drinking, comorbidities (diabetes mellitus, hypertension, coronary artery diseases) and stage of malignant diseases

### Subgroup analysis

**Fig. 4:**
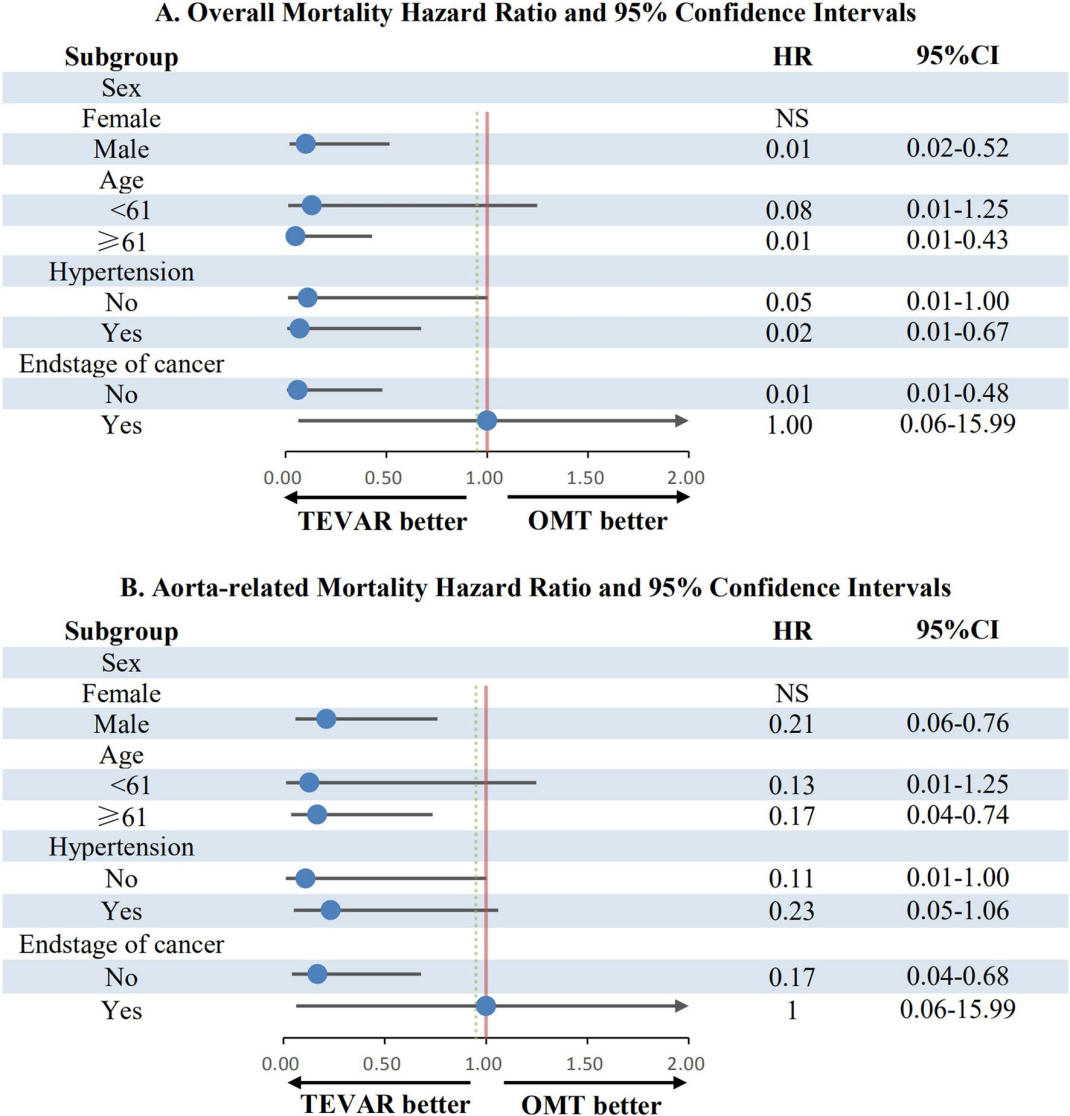
Subgroup analysis: HRs for overall mortality **A** and aorta-related mortality (**B**)

Subgroup analysis was conducted to explore the association between TEVAR or OMT and the 5 year overall or aortic-disease related mortality in the cohort stratified by age, sex, hypertension status, and the stage of malignancies (Fig. 4). Patients ≥ 61 years of age and patients < 61 years exhibited comparable risk reduction with respect to overall mortality (HR, 0.01; 95% CI, 0.01–0.43 versus HR, 0.08; 95% CI, 0.01–1.25) when receiving TEVAR versus the OMT group (Fig. 4A). In terms of aortic-disease related mortality, patients aged ≥ 61 years had a similarly low risk (HR, 0.17; 95% CI, 0.04–0.74) compared to patients < 61 years (HR, 0.13; 95% CI, 0.01–1.25) when receiving TEVAR versus the OMT group (Fig. 4B). In the male sex subgroup, patients receiving TEVAR showed significant risk reduction with respect to overall mortality (HR, 0.01; 95% CI, 0.02–0.52) and aortic-disease related mortality (HR, 0.21; 95% CI, 0.06–0.76), similar to the entire cohort. Patients with hypertension (HR, 0.02; 95% CI, 0.01–0.67) or without hypertension (HR, 0.05; 95% CI, 0.01–1.00) showed significant risk reduction with respect to overall mortality upon receiving TEVAR, similar to the entire cohort (Fig. 4B). Furthermore, there was also a significant risk reduction with respect to aortic-disease related mortality upon receiving TEVAR, similar to the entire cohort in patients with hypertension (HR, 0.23; 95% CI, 0.05–1.06) or without hypertension (HR, 0.11; 95% CI, 0.01–1.00). However, the sample size of female patients (n = 4) and patients with end-stage diseases (n = 4) was small in the Cox model, making stratified analysis impossible (Fig. 4).

## Discussion

In this work, we show that compared to OMT, TEVAR results in a better prognosis for ATBAD patients with a single tumor type. In particular, the risk of overall mortality and aortic-disease related mortality was lower in the TEVAR group than in the OMT group.

To date, there have been no studies or conclusions on the prognosis of ATBAD patients with tumors treated using OMT and TEVAR. However, there is no doubt that AD and tumors share some traditional risk factors, among which cytokines are the most prominent. [6, 7, 11] Therefore, we believe that tumors modulate the effect of OMT and TEVAR on the prognosis of patients with ATBAD. In the present study, through Kaplan‒Meier survival analysis, we found that compared to the OMT group, the TEVAR group had better 5 year overall survival (Fig. 2) and 5 year aortic-disease related survival rates (Fig. 3). To our knowledge, this is the first study on the association between the outcomes of OMT and TEVAR in ATBAD patients with a single tumor type. Further, these data provide some evidence that TEVAR is more effective than OMT in the treatment of these patients. However, there are still limitations regarding the number of cases analyzed, and expansion of the sample size is required. Importantly, the complex pathological process of AD is closely related to the infiltration of inflammatory cells into the aortic wall. [6] TEVAR is widely employed among ATBAD patients, because it is easy and effective, and it also plays a role in improving the aortic wall. [12] In such patients, TEVAR treatment may help reduce the release of AD-related cytokines and inhibit tumor growth to some extent.

The Cox proportional hazard model with unadjusted risk showed that the TEVAR group had a lower 5 year overall mortality (HR, 0.17; 95% CI, 0.05–0.56) and aortic-disease related risk (HR, 0.08; 95% CI, 0.02–0.39) than the OMT group. However, age and male sex are also risk factors for AD. [13] In further Cox proportional hazard analysis in age- and sex-adjusted models, the hazard of 5 year overall mortality (HR, 0.18; 95% CI, 0.05–0.61) and aortic-disease related mortality (HR, 0.18; 95% CI, 0.02–0.41) were consistently lower in the TEVAR group than in the OMT group. A meta-analysis showed that there is a negative correlation between diabetes mellitus and AD. [14] Compared to ATAAD, ATBAD has a significantly higher correlation with coronary artery diseases [15]. In addition, smoking, drinking, and hypertension also influence the clinical features of AD. [16] In the sex-, smoking-, drinking-, and comorbidities (diabetes mellitus, hypertension, and coronary artery diseases)-adjusted model, the hazard ratio of 5 year overall mortality was 78.0% lower (HR, 0.22; 95% CI, 0.06–0.81), and the risk of aortic-disease related mortality was 93.0% lower (HR, 0.07; 95% CI, 0.01–0.61) for the TEVAR group relative to that for the OMT group. It can be concluded from the above results that, despite suffering from a single malignant tumor, patients with ATBAD may still benefit from TEVAR; however, due to the small sample size these results need to be verified using a larger cohort in the future in order to conduct a prospective cohort study of TEVAR and ATBAD with a single tumor.

The mean age of AD prevalence is ~ 61 years [8], and subgroup analysis was conducted to explore the effect of TEVAR or OMT on the ATBAD population with a single tumor stratified by age. Patients ≥ 61 years of age had comparable risk reduction in overall mortality (HR, 0.01; 95% CI, 0.01–0.43) and aortic-disease related mortality (HR, 0.17; 95% CI, 0.04–0.74) compared to the overall mortality (HR, 0.08; 95% CI, 0.01–1.25) and aortic-disease related mortality (HR, 0.13; 95% CI, 0.01–1.25) of those aged < 61 years when receiving TEVAR versus the OMT group. Men are one of the greatest population risks of AD [17]. In the male sex subgroup, compared to the OMT group, ATBAD patients with a single tumor who underwent TEVAR showed significant risk reduction in 5 year overall mortality (HR, 0.01; 95% CI, 0.02–0.52) and aortic-disease related mortality (HR, 0.21; 95% CI, 0.06–0.76), similar to the entire cohort. However, hypertension (HR, 0.02; 95% CI, 0.01–0.67) or no-hypertension (HR, 0.05; 95% CI, 0.01–1.00) status of ATBAD patients with a single tumor showed significant risk reduction in the 5 year overall mortality for the TEVAR group versus the OMT group. We initially concluded that compared to OMT, TEVAR significantly reduces the prognostic risk of the ATBAD population with a single tumor and is not affected by age or sex. However, sample limitations still exist, and the sample needs to be further expanded. Nevertheless, our results indicate to some extent that TEVAR is a better choice for ATBAD patients with a single tumor.

The strengths of this study are as follows: (1) we verified the clinical value of TEVAR, which may help clinicians make clinical decisions for such patients; (2) considering the fact that cases of ATBAD with a single tumor are rare, the sample size of this study is relatively large; (3) the prognostic association between TEVAR and the ATBAD population with a single tumor has been fully confirmed in this study, and many factors such as sex, smoking, drinking, and comorbidities (diabetes mellitus, hypertension, and coronary artery diseases) have been adjusted. However, this study has several limitations. First, there were only 40 ATBAD population samples with complicated tumors. It is difficult to analyze subgroups according to different tumor types and therapy methods, and these heterogeneities may affect the results of the study. Second, as there are fewer patient sub-categories based on parameters, such as sex, age, smoking and drinking, and other comorbidities, it is impossible to derive a clear causal relationship from this cross-sectional design; therefore, more prospective cohort studies are needed in the future. In addition, the impact of TEVAR on the prognosis of ATBAD in a population displaying different tumor stages has not been determined. Moreover, the prognostic difference between OMT and TEVAR for ATBAD with or without hypertension populations is unknown.

## Conclusions

Despite suffering from a single malignant tumor, patients with ATBAD may still benefit from TEVAR. This study provides evidence for clinical treatment and raises additional questions regarding clinical research in this population.

## Data Availability

The clinical data supporting the conclusion of this study were derived from the paper and electronic records of patients. Due to data protection regulation, anonymized data are available from the corresponding author on reasonable request and with the permission of the ethical committee of Changhai Hospital.

## Abbreviations

TEVAR: Thoracic endovascular aortic repair
ATBAD: Acute type B aortic dissection
OMT: Optimal medical treatment
HR: Hazard ratio
CI: Confidence interval
AD: Aortic dissection
IRAD: International Register of Acute Aortic Dissection
